# Transportation of AIE-visualized nanoliposomes is dominated by the protein corona

**DOI:** 10.1093/nsr/nwab068

**Published:** 2021-04-24

**Authors:** Yi-Feng Wang, Chunqiu Zhang, Keni Yang, Yufei Wang, Shaobo Shan, Yan Yan, Kenneth A Dawson, Chen Wang, Xing-Jie Liang

**Affiliations:** CAS Key Laboratory for Biomedical Effects of Nanomaterials and Nanosafety, CAS Center for Excellence in Nanoscience, National Center for Nanoscience and Technology of China, Beijing 100190, China; University of Chinese Academy of Sciences, Beijing 100049, China; CAS Key Laboratory for Biomedical Effects of Nanomaterials and Nanosafety, CAS Center for Excellence in Nanoscience, National Center for Nanoscience and Technology of China, Beijing 100190, China; State Key Laboratory of Medicinal Chemical Biology, Key Laboratory of Bioactive Materials of Ministry of Education, and College of Life Sciences, Nankai University, Tianjin 300071, China; CAS Key Laboratory for Biomedical Effects of Nanomaterials and Nanosafety, CAS Center for Excellence in Nanoscience, National Center for Nanoscience and Technology of China, Beijing 100190, China; CAS Key Laboratory for Biomedical Effects of Nanomaterials and Nanosafety, CAS Center for Excellence in Nanoscience, National Center for Nanoscience and Technology of China, Beijing 100190, China; University of Chinese Academy of Sciences, Beijing 100049, China; CAS Key Laboratory for Biomedical Effects of Nanomaterials and Nanosafety, CAS Center for Excellence in Nanoscience, National Center for Nanoscience and Technology of China, Beijing 100190, China; Centre for BioNano Interactions, School of Chemistry, University College Dublin, Dublin 4 D04 V1W8, Ireland; School of Biomolecular and Biomedical Science, University College Dublin, Dublin 4 D04 V1W8, Ireland; Guangdong Provincial Education Department Key Laboratory of Nano-Immuno-regulation Tumor Microenvironment, The Second Affiliated Hospital, Guangzhou Medical University, Guangzhou 510260, China; Centre for BioNano Interactions, School of Chemistry, University College Dublin, Dublin 4 D04 V1W8, Ireland; CAS Key Laboratory for Biomedical Effects of Nanomaterials and Nanosafety, CAS Center for Excellence in Nanoscience, National Center for Nanoscience and Technology of China, Beijing 100190, China; University of Chinese Academy of Sciences, Beijing 100049, China; CAS Key Laboratory for Biomedical Effects of Nanomaterials and Nanosafety, CAS Center for Excellence in Nanoscience, National Center for Nanoscience and Technology of China, Beijing 100190, China; University of Chinese Academy of Sciences, Beijing 100049, China

**Keywords:** aggregation-induced emission (AIE), cationic liposomes, protein corona, endocytosis, membrane fusion

## Abstract

Liposomes, especially cationic liposomes, are the most common and well-investigated nanocarriers for biomedical applications, such as drug and gene delivery. Like other types of nanomaterials, once liposomes are incubated in a biological milieu, their surface can be immediately cloaked by biological components to form a protein corona, which confers a new ‘biological identity’ and modulates downstream interactions with cells. However, it remains unclear how the protein corona affects the transportation mechanism after liposomes interact with cells. Here, we employed home-made aggregation-induced-emission-visualized nanoliposomes TR4@Lipo as a model to investigate transportation with or without the protein corona by optical imaging techniques. The results show that the protein corona can change the cellular transportation mechanism of TR4@Lipo from energy-independent membrane fusion to energy-dependent endocytosis. The protein corona also modulates the intracellular distribution of loaded cargoes. This knowledge furthers our understanding of bio-nano interactions and is important for the efficient use of cationic liposomes.

## Teaser

Using home-made AIE-visualized nanoliposomes TR4@Lipo as a model, this work reports that the protein corona can change the cellular transportation mechanism of TR4@Lipo from energy-independent membrane fusion to energy-dependent endocytosis.

## INTRODUCTION

Liposomes are a versatile gene/drug delivery system with numerous potential applications in biomedicine due to their special structure, with a hydrophobic bilayer and an aqueous core for the storage of hydrophobic or hydrophilic contents, and their biocompatibility and biodegradability [1–3]. Among the different types of liposomes, cationic liposomal formulations have attracted a lot of attention because of their capacity to bind negatively charged nucleic acids and perform as non-viral gene delivery systems [4,5]. More importantly, many cationic liposomes have been reported to fuse with cell plasma membranes, which can lead to direct cargo release from liposomes into the cytoplasm and thus the enhancement of drug delivery speed and efficiency [6–8]. For instance, Csiszar *et al.* reported that liposomes composed of cationic lipids and aromatic molecules (e.g. fluorescent dye) can achieve significant fusion efficiency with living cells within a few minutes [9].

Currently, in many examples, the efficiency of cargo or gene delivery by cationic liposomes is evaluated *in vitro* in a serum-free environment, which is partially because lipid-based vectors are prone to aggregation induced by proteins and the delivery efficiency is reduced in serum-containing medium [10–13]. However, serum proteins and biomolecules cannot be avoided in an *in vivo* system; this means that the serum-free condition cannot represent the real performance of liposomes in a biological environment. It is known that in biological fluids, nanomaterials such as liposomes can interact with proteins and other biomolecules, forming a layer known as the protein corona [14–16]. Thus, the fate of liposomes could be different from the original expectation after corona formation because the physicochemical properties are changed [16,17]. Therefore, in order to fully understand the transportation behavior of cationic liposomes in a biological environment and thus guide the design of efficient liposomal formulations, the effect of protein corona formation on their interaction with cells should be studied.

Optical imaging, with its high spatiotemporal resolution and superb sensitivity, offers a convenient way to visualize biological events and plays an essential role in basic biological research [18,19]. Thus, in this context, in order to investigate the effect of the protein corona on the interaction of cationic liposomes with cells in a visible way, we introduced a positively charged cell membrane probe (TR4) into anionic liposomes (hydrogenated phosphatidylcholine (HSPC), cholesterol and DSPE-PEG2000). TR4 contains four arginine residues, a palmitic acid tail and tetraphenylethylene (TPE). This structure combines multiple functions into one molecule: (i) the hydrophilic arginine residues make TR4 water soluble, and their positively charged side chains can convert the anionic liposomes into cationic liposomes; (ii) the palmitic acid tail anchors TR4 into lipid bilayers; (iii) TPE is a typical fluorophore with the aggregation-induced emission (AIE) property [20,21], first reported by Tang *et al.*, and is a potential initiator for membrane fusion, based on the principle that combining a cationic lipid and an aromatic molecule can increase the fusion ability of liposomes [11]. More importantly, the fluorescence intensity of TR4 is dramatically enhanced when it is inserted into lipid bilayers because of the restriction of intermolecular rotation (RIR) effect, as reported in our previous work [22]. This endows TR4-containing liposomes (TR4@Lipo) with a self-indicating property that can be monitored by confocal microscopy.

In this way, taking advantage of the cell membrane probe TR4, we constructed cationic liposomes and visualized their interactions with cells by microscopy in serum-free conditions and standard cell culture conditions. This allowed us to study the effects of protein corona formation on the cellular transportation of cationic liposomes in a biologically relevant environment.

## RESULTS AND DISCUSSION

### Characterization of TR4@Lipo and their uptake pathway in the absence of protein corona

**Figure 1.**
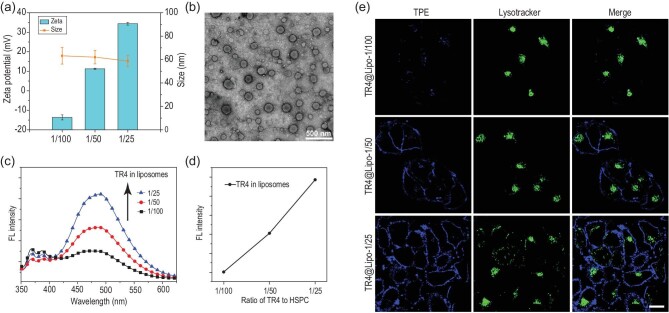
(a) Size and zeta potential of TR4@Lipo with the different ratios of TR4 (from 1/100 to 1/25). (b) Transmission electron microscopy image of TR4@Lipo (the ratio of TR4 is 1/25). (c) Fluorescence spectra of TR4@Lipo with the different ratios of TR4 (from 1/100 to 1/25). The excitation wavelength is 330 nm. (d) Fluorescence intensity of TR4@Lipo with the different ratios of TR4 (from 1/100 to 1/25) at 466 nm. (e) Confocal images of MCF-7 cells after treatment with TR4@Lipo (with the different ratios of TR4) in the absence of FBS. The blue color is from TR4 (λ_ex_ = 405 nm). The green color is from LysoTracker Deep Red (λ_ex_ = 630 nm). Scale bar is 10 μm.

The AIE-visualized cationic liposomes TR4@Lipo were made by the co-assembly of TR4, HSPC, cholesterol (which stabilizes the liposomes) and DSPE-PEG2000 (which increases the dispersibility and prevents the aggregation of liposomes, Supplementary Fig. 1). The ratio of TR4 to HSPC was varied from 1/100 to 1/25. TR4@Lipo with different ratios of TR4 were characterized by dynamic light scattering (DLS) and zeta potential measurements. These showed that as the ratio of TR4 increased in the liposomes, the surface positive charge increased because more arginine residues were exposed on the surface, but there was almost no change in the diameter of the liposomes (Fig. 1a). Even at the highest ratio of TR4 to HSPC (1/25), the liposome morphology was still maintained (Fig. 1b). In our previous work, we showed that no fluorescence was observed when the TR4 existed in the monomer state in aqueous solution [22]. Once TR4 molecules were inserted into liposomes, dramatic ‘turn-on’ fluorescence occurred. The intramolecular rotation of TPE would be restricted by the tangle of lipids, thus activating the AIE property of TPE [23]. Here, we found that the fluorescence intensities were also increased when the ratio of TR4 to HSPC increased from 1/100 to 1/25 (Fig. 1c and d). This suggests that the fluorescent cationic liposome TR4@Lipo will potentially be visible for tracking the transportation behavior in living cells.

In order to test the fluorescence property of TR4@Lipo for visually tracking liposomes in living cells, MCF-7 cells were treated with TR4@Lipo in serum-free conditions and then imaged by laser scanning confocal microscopy. The same concentration of TR4@Lipo was used for each treatment, but the ratio of TR4 varied. As shown in Fig. 1e, the fluorescence intensities of TR4@Lipo increased with an increased ratio of TR4 in the liposomes, which is consistent with the fluorescence spectrum results. As expected, TR4@Lipo with the highest ratio of TR4 exhibited the highest positive charge and the brightest fluorescence intensity, while the cellular toxicity was comparable to liposomes with TR4 to HSPC ratios of 1/50 and 1/100 [22]. The ratio was then fixed at 1/25 for the following studies (Supplementary Fig. 2a and Table 1). Importantly, we noticed an interesting phenomenon that the fluorescence from TR4@Lipo (blue color) was mainly localized on the cell membrane and there was no overlap with lysosomes (green color). We speculated that TR4@Lipo may fuse with the cell membrane, similar to the previously reported fusogenic liposomes [24,25].

### Comparison of the protein corona composition between TR4@Lipo and normal liposomes

**Figure 2.**
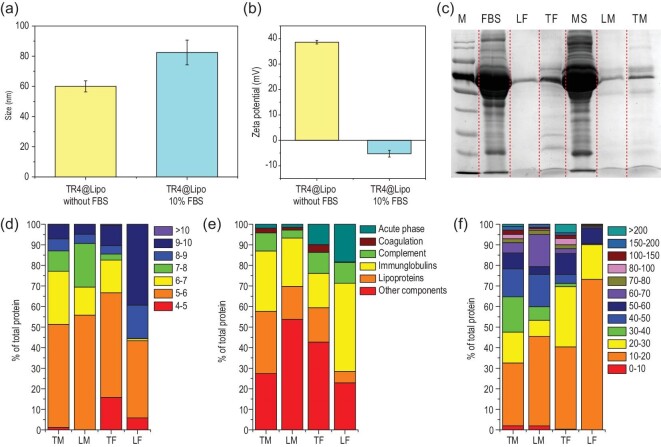
(a) Size and (b) zeta potential of TR4@Lipo in the presence or absence of 10% FBS. (c) Sodium dodecyl sulfate-polyacrylamide gel electrophoresis (SDS-PAGE) analysis of corona proteins recovered from TR4@Lipo and normal Lipo. M, protein size marker; FBS, fetal bovine serum; LF, normal Lipo incubated with 10% FBS; TF, TR4@Lipo incubated with 10% FBS; MS, mouse serum; LM, normal Lipo incubated with 10% mouse serum; TM, TR4@Lipo incubated with 10% mouse serum. (d–f) The identified proteins in the corona were classified based on (d) isoelectric point, (e) biological processes and (f) calculated molecular mass. TM, TR4@Lipo incubated with 10% mouse serum; LM, normal Lipo incubated with 10% mouse serum; TF, TR4@Lipo incubated with 10% FBS; LF, normal Lipo incubated with 10% FBS.

Once nanostructures are incubated in a biological milieu, their surface can become immediately cloaked by biological components to form a protein corona, which confers a new ‘biological identity’ on the nanostructures. It is known that a close membranous contact between liposomes and cells is essential for membrane fusion [26]. Thus, we questioned whether the formation of a protein corona on the surface of TR4@Lipo affects their interaction with cells and alters the membrane fusion behavior as observed in the serum-free condition. To answer this question, the protein corona on the TR4@Lipo surface was characterized. Liposomes without TR4 modification (normal Lipo) were used as a comparison (Supplementary Fig. 2b and Table 1). The TR4@Lipo are positively charged and the zeta potential is over 30 mV, while the normal Lipo has a negative charge with a zeta potential around −30 mV. The TR4@Lipo were incubated with 10% fetal bovine serum (FBS, a standard serum concentration for cell culture) and the physicochemical properties were then characterized by DLS and zeta potential measurement. As shown in Fig. 2a and b, we found that the size of TR4@Lipo reached around 80 nm after serum exposure, which was 20 nm thicker than the naked TR4@Lipo, and the zeta potential was totally reversed from positive to negative. However, the change of size and zeta potential of normal Lipo was negligible (Supplementary Fig. 3). The results indicate that the natural physicochemical properties of the TR4@Lipo surface can be changed by the protein corona. The liposome-corona complexes were then isolated from the serum and characterized by gel electrophoresis. The results showed that after the incubation with 10% FBS, only a few protein bands were detected in the normal Lipo lane, but more were detected in the TR4@Lipo lane, which is in agreement with the size and zeta potential analysis (Fig. 2c). The same tendency was also observed after the TR4@Lipo and normal Lipo were incubated with mouse serum, which suggests that the protein adsorption capacity of TR4@Lipo is higher than that of normal Lipo.

Next, the protein corona compositions on different liposomes were identified using label-free quantitative liquid chromatography mass spectrometry. The semi-quantitative analysis showed that the total amount of protein adsorbed by TR4@Lipo is 28.5-fold and 27.2-fold higher than that of normal Lipo after treatment with 10% FBS and mouse serum, respectively. For normal Lipo, 31 proteins were identified after incubation with 10% FBS. However, for TR4@Lipo, 308 proteins were identified after incubation with 10% FBS. Similar results were also obtained when the liposomes were exposed to mouse serum: 543 proteins were detected in the TR4@Lipo group while 100 proteins were detected in the normal Lipo group. To further identify the biological relevance of the corona composition, the isoelectric point, molecular mass and biological process were analyzed for all the adsorbed proteins in each group, as shown in Fig. 2d–f. The top 20 most abundant proteins are listed in Supplementary Table 2. The analysis showed that proteins with isoelectric point (pI) < 7 were enriched on TR4@Lipo and normal Lipo, irrespective of negative or positive charge on the liposome surface, consistent with previous studies (Fig. 2d) [27,28]. It is possible that the surface charge is critical for deciding the amount of adsorbed protein on the surface [29]. In addition, we observed that the percentage of immunoglobulins, complement proteins and lipoproteins is high in both the 10% FBS and mouse serum groups (Fig. 2e). These proteins provide insight into the potential biological processes that could be activated after treatment with liposomes *in vitro* or *in vivo*, as these proteins to some extent represent the real biological identity of the liposomes. We further noticed that small molecular mass proteins (<60 kDa) constitute the majority of the corona in TR4@Lipo and normal Lipo (Fig. 2f). The distribution of molecular weights of the corona proteins is comparable to the distribution in native serum (Fig. 2c) [30].

### The transportation mechanisms of TR4@Lipo are mediated by the protein corona

**Figure 3.**
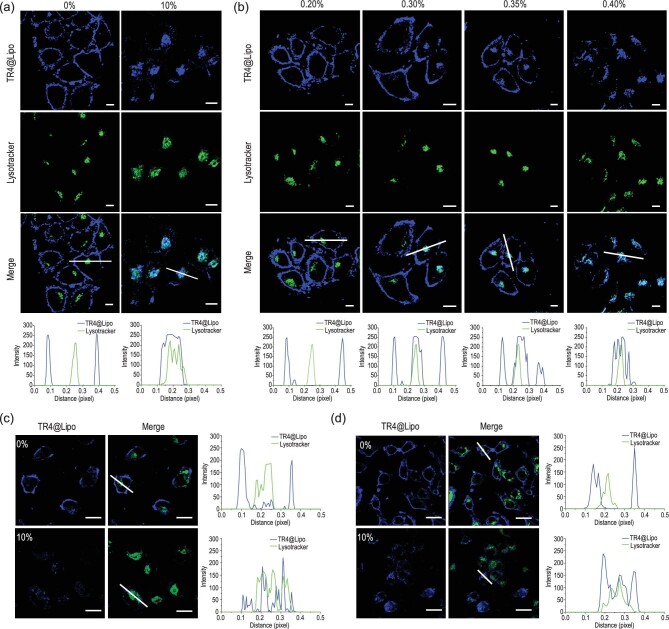
(a) Confocal images of MCF-7 cells after treatment with TR4@Lipo in the presence or absence of 10% FBS. Scale bar is 10 μm. Co-localization profiles are shown underneath and were analyzed along the white lines in the ‘Merge’ images. (b) Confocal images of MCF-7 cells after treatment with TR4@Lipo in the presence of different concentrations of FBS from 0.2% to 0.4%. Scale bar is 10 μm. Co-localization profiles are shown underneath. (c and d) Confocal images of (c) A549 and (d) NRK cells after treatment with TR4@Lipo in 10% FBS. Scale bar is 20 μm. Co-localization profiles are shown on the right. The blue color is from TR4 (λ_ex_ = 405 nm). The green color is from LysoTracker Deep Red (λ_ex_ = 630 nm). ImageJ software was used for co-localization analysis.

After confirming the higher protein adsorption capacity of TR4@Lipo compared to normal Lipo, we then tested whether formation of the protein corona would affect the transportation mechanism of TR4@Lipo at the cellular level. As shown in Fig. 3a, TR4@Lipo were localized on the cell membrane in the absence of FBS, but they co-localized with lysosomes in the presence of 10% FBS. This indicates that formation of the protein corona changed the transportation behavior of TR4@Lipo. It is possible that the protein corona, which confers a new biological identity on TR4@Lipo, is recognized by the cell membrane and consequently the membrane fusion property of TR4@Lipo is lost.

To determine the critical concentration of serum protein for switching the transportation behavior of TR4@Lipo, we incubated TR4@Lipo with gradually increasing concentrations of FBS and measured the zeta potential. As shown in Supplementary Fig. 4a, we found that as the concentration of serum increased from 0.0% to 0.6%, the zeta potential of TR4@Lipo gradually changed from positive to negative. The critical concentration of serum, at which TR4@Lipo were neutral, is around 0.3%. In addition, non-denaturing gel electrophoresis was employed to further confirm the critical concentration of serum. We reasoned that if the concentration of FBS did not saturate the surface of liposomes, we would see a different protein migration pattern compared to the serum itself. If the liposome surface was saturated, some free serum protein would appear. The image in Supplementary Fig. 4b demonstrates that the TR4@Lipo could not bind more proteins on their surface when the concentration of serum was over 0.3%, consistent with the zeta potential analysis results.

Next, the transportation behavior of TR4@Lipo was investigated in the presence of different concentrations of FBS (from 0.2 to 0.4%). As shown in Fig. 3b, we found that in 0.2% FBS, TR4@Lipo were located on the cell membrane. Once the concentration of FBS was increased to 0.3%, most of the TR4@Lipo were still on the cell membrane, but some were co-localized with the lysosomes, which suggests that the transportation mechanism of some TR4@Lipo has been changed. In 0.35% FBS, some TR4@Lipo are still in the plasma membrane, but in 0.4% FBS, almost all of the TR4@Lipo co-localize with lysosomes. This indicates that the transportation mechanism of TR4@Lipo has been changed from membrane fusion to endocytosis. As the cell membrane composition is heterogeneous and changes with different cell types [31,32], this phenomenon was further checked in the human adenocarcinoma cell line A549, the normal rat kidney cell line NRK, the human triple-negative breast cancer cell line MDA-MB-231 and the normal mouse muscle cell line C2C12. As expected, for all four cell lines, the TR4@Lipo co-localized with the cell membrane in the absence of FBS, and TR4@Lipo co-localized with lysosomes in 10% FBS (Fig. 3c and d, and Supplementary Fig. 5). A further consideration is that different types of protein in the biological milieu may affect the composition of the protein corona on the surface of TR4@Lipo, and the new biological identity may further affect the transportation behavior of TR4@Lipo. To address this, we tested the transportation behavior of TR4@Lipo in the presence of human serum album (HSA) and human serum (HS). As shown in Supplementary Fig. 6, the TR4@Lipo co-localized with lysosomes after exposure to 10% HSA or 10% HS, which is highly consistent with the behavior in the presence of 10% FBS. These results indicate that the transformation of transportation behavior from membrane fusion to endocytosis is universal.

**Figure 4.**
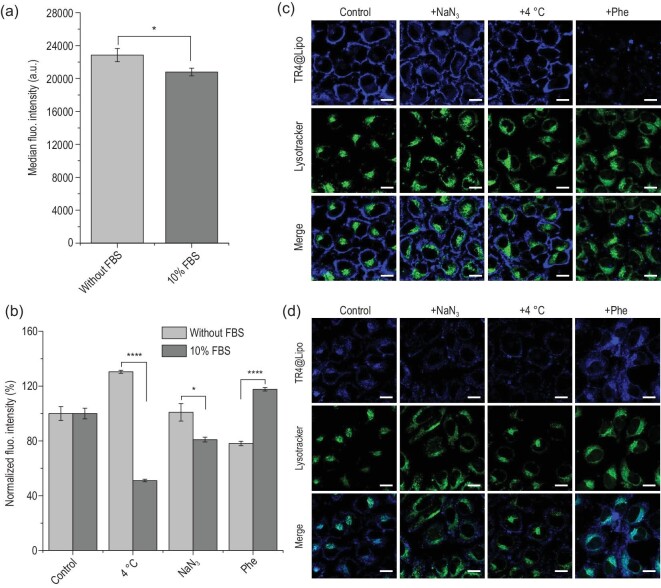
(a) Uptake of TR4@Lipo by MCF-7 cells in the absence or presence of FBS at 37°C for 4 h. Datasets were compared by unpaired t-test to identify significant differences. ^∗^, p < 0.05; ^∗∗∗∗^, p < 0.0001. (b) Uptake of TR4@Lipo by MCF-7 cells in the absence or presence of FBS for 4 h under the following conditions: 4°C; NaN_3_ at 37°C; Z-Phe-Phe-Phe-OH (Phe) at 37°C. The uptakes were normalized against the uptake in cells without inhibition conditions to show the inhibition efficacy. Datasets were compared by unpaired t-test to identify significant differences. ^∗^, p < 0.05; ^∗∗∗∗^, p < 0.0001. (c and d) Confocal images of TR4@Lipo uptake by MCF-7 cells treated with different inhibition conditions, (c) in the absence of FBS and (d) in the presence of 10% FBS. The blue color is from TR4 (λ_ex_ = 405 nm) and the green color is from LysoTracker Deep Red (λ_ex_ = 630 nm). Scale bar is 20 μm.

Electrostatic interactions with negatively charged cell membranes may contribute to the adsorption of TR4@Lipo onto the cell membrane [33]. In order to confirm the fusion behavior of TR4@Lipo with cell membranes and exclude the possibility of liposome attachment to the cell surface, we first made a simple fluorescence resonance energy transfer (FRET) model to prove the fusion property of TR4@Lipo (Supplementary Fig. 7a). FRET is a process in which the energy from the excited state of a donor molecule is transferred to an acceptor molecule. For FRET to occur, the distance between the donor and acceptor is limited to a maximum of ∼10 nm [34,35]. In our previous work, we reported that FRET happens when TPE and the small molecule drug DOX are mixed or conjugated together [36,37]. Therefore, we created DOX-loaded normal Lipo (DOX@Lipo) to mimic the cell membrane, and we mixed them with TR4@Lipo while monitoring the fluorescence intensity to check for FRET. After mixing, the fluorescence intensity of TR4 at 466 nm was decreased and the intensity of DOX at 590 nm was increased, which suggests that the two molecules were sufficiently close for energy transfer from TR4 to DOX (Supplementary Fig. 7b). This result indicates that TR4@Lipo have the membrane fusion property. Subsequently, experiments were carried out *in vitro* using inhibitors of different transportation pathways, followed by flow cytometry and confocal microscopy to test the fusion property of TR4@Lipo in living cells. Before analysis with inhibitors, MCF-7 cells were treated with TR4@Lipo in the absence or presence of FBS at 37°C and then analyzed by flow cytometry as a control. As shown in Fig. 4a, the median fluorescence intensity of TR4@Lipo in the absence of FBS is higher than that in the presence of FBS, suggesting that the uptake efficiency of TR4@Lipo in the absence of FBS is higher than in the presence of FBS. This is probably because FBS changed the surface charge of TR4@Lipo from positive to negative, thus weakening the interaction between TR4@Lipo and the negatively charged cell membrane. Previous studies have shown that the energy-dependent endocytosis process can be blocked by reducing the culture temperature or depleting cellular energy [38,39]. Therefore, MCF-7 cells were treated with TR4@Lipo in the absence or presence of FBS under different inhibition conditions, i.e. 4 h at 4°C, or 4 h at 37°C with sodium azide, which depletes the energy for endocytosis. After normalizing the fluorescence intensity with the control group, the normalized fluorescence intensity of TR4@Lipo in the absence of FBS was weakly increased, but the fluorescence intensity in the presence of FBS was obviously reduced at low temperature (Fig. 4b). Similarly, the normalized fluorescence intensity of TR4@Lipo in the absence of FBS was almost unchanged, but the fluorescence intensity in the presence of FBS was reduced after treatment with sodium azide. These results indicate that the transportation of TR4@Lipo is an energy-independent process in the absence of FBS and is an energy-dependent process in the presence of FBS. Next, a fusion inhibitor peptide analog Z-Phe-Phe-Phe-OH was used to inhibit the membrane fusion process [40,41]. The normalized fluorescence intensity of cells treated with TR4@Lipo was obviously reduced in the absence of FBS but was weakly increased in the presence of FBS, which suggests that the TR4@Lipo interact with cells in the absence of FBS through membrane fusion. The same inhibitory effects were further observed by confocal imaging in the absence of FBS (Fig 4c) and in the presence of FBS (Fig. 4d). In order to further confirm the effect of the protein corona on the transportation mechanisms, three common small molecule inhibitors of endocytosis were used, including chlorpromazine (12.5  μg/mL, an inhibitor of clathrin-mediated endocytosis), cytochalasin D (5  μg/mL, an inhibitor of actin polymerization) and methyl-β-cyclodextrin (M-β-CD, 5 μg/mL, an inhibitor of caveola-mediated endocytosis) [38,42]. As shown in Supplementary Fig. 8, compared with the control group, the three inhibitors exerted minor inhibition effects in the absence of FBS. However, in the presence of FBS, the normalized fluorescence intensities of cells treated with TR4@Lipo were lower than in the absence of FBS, particularly under treatment with cytochalasin D and M-β-CD. This indicates that the internalization of TR4@Lipo occurs through the energy-dependent endocytosis pathway in the presence of FBS. Among the inhibitors, the greatest uptake reduction (up to 42%) was seen for M-β-CD, which possibly suggests that the caveola-mediated endocytosis pathway is the main route for the endocytosis of TR4@Lipo in the presence of FBS. Collectively, our analyses strongly suggest that TR4@Lipo can fuse with cell membranes in the absence of FBS, but after formation of the protein corona on the surface of TR4@Lipo, TR4@Lipo are recognized and taken up by cells through energy-dependent endocytosis. These results provide evidence that TR4@Lipo, as a kind of fusogenic liposome, may be a promising system for efficient intracellular delivery in serum-free conditions.

### The protein corona modulates the intracellular distribution of cargoes

**Figure 5.**
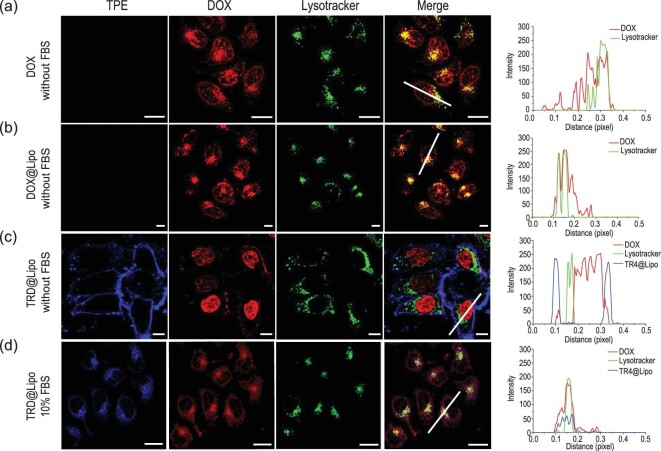
(a) Confocal images of MCF-7 cells showing the subcellular distributions of (a) free DOX, (b) DOX@Lipo, (c) TRD@Lipo in the absence of FBS and (d) TRD@Lipo in the presence of 10% FBS. Co-localization profiles are shown on the right. Fluorescence intensity was analyzed along the white lines in the ‘Merge’ images. The blue color is from TR4 (λ_ex_ = 405 nm), the red color is from DOX (λ_ex_ = 488 nm) and the green color is from LysoTracker Deep Red (λ_ex_ = 630 nm). Scale bar is 10 μm. ImageJ software was used for co-localization analysis.

The transportation mechanisms of TR4@Lipo are altered by formation of the protein corona, so next we investigated whether the protein corona would further modulate the delivery and release of cargoes in TR4@Lipo. Here, the small molecule drug DOX was chosen as the model cargo. DOX has been widely used as the cargo for different delivery systems [43–45]. The DOX was encapsulated into TR4@Lipo (TRD@Lipo) and normal Lipo (DOX@Lipo). Size and zeta potential analysis showed that the physicochemical properties of TRD@Lipo and DOX@Lipo are similar to the empty liposomes (Supplementary Fig. 9 and Table 1). Then, MCF-7 cells were incubated with free DOX and the different DOX/liposome formulations and the intracellular distributions of DOX were studied. In the cells treated with free DOX, most of the DOX molecules were located in the nuclear region while some of them merged with lysosomes (Fig. 5a). This corresponds to reports in the literature that free DOX is indiscriminately located within other organelles, such as lysosomes and mitochondria [46]. Compared to free DOX, DOX encapsulated in the DOX@Lipo was mainly co-localized with lysosomes and the nucleus in the absence of FBS, which indicates that the DOX@Lipo was internalized through the endocytosis pathway into lysosomes (Fig. 5b). For TRD@Lipo in the absence of 10% FBS, fusion with the cell membrane was observed, similar to TR4@Lipo. Some of the DOX signal was on the cell membrane, merged with the TR4 fluorescence. Inside the cell, the DOX was mainly located in the nuclear region and there was no overlap with lysosomes or other organelles (Fig. 5c). These results indicate that the TRD@Lipo still utilized the membrane fusion pathway to deliver DOX into the nucleus.

In contrast, for TRD@Lipo in the presence of 10% FBS, most of the DOX and TR4 signals co-localized well together with the lysosomes (Fig. 5d). This reveals that TRD@Lipo was internalized through the endocytosis pathway into lysosomes. We further tested whether endocytosis still occurred after the TRD@Lipo was incubated with 10% FBS first, and the free FBS was removed before incubation with cells. As expected, most of the TRD@Lipo was still co-localized with lysosomes, similar to the results when TRD@Lipo was incubated with cells in the presence of 10% FBS (Supplementary Fig. 10). Collectively, these results demonstrate that when the transportation mechanism of TR4@Lipo is switched by the protein corona from membrane fusion to endocytosis, the intracellular distribution of cargoes can also be changed.

## CONCLUSION

**Scheme 1.**
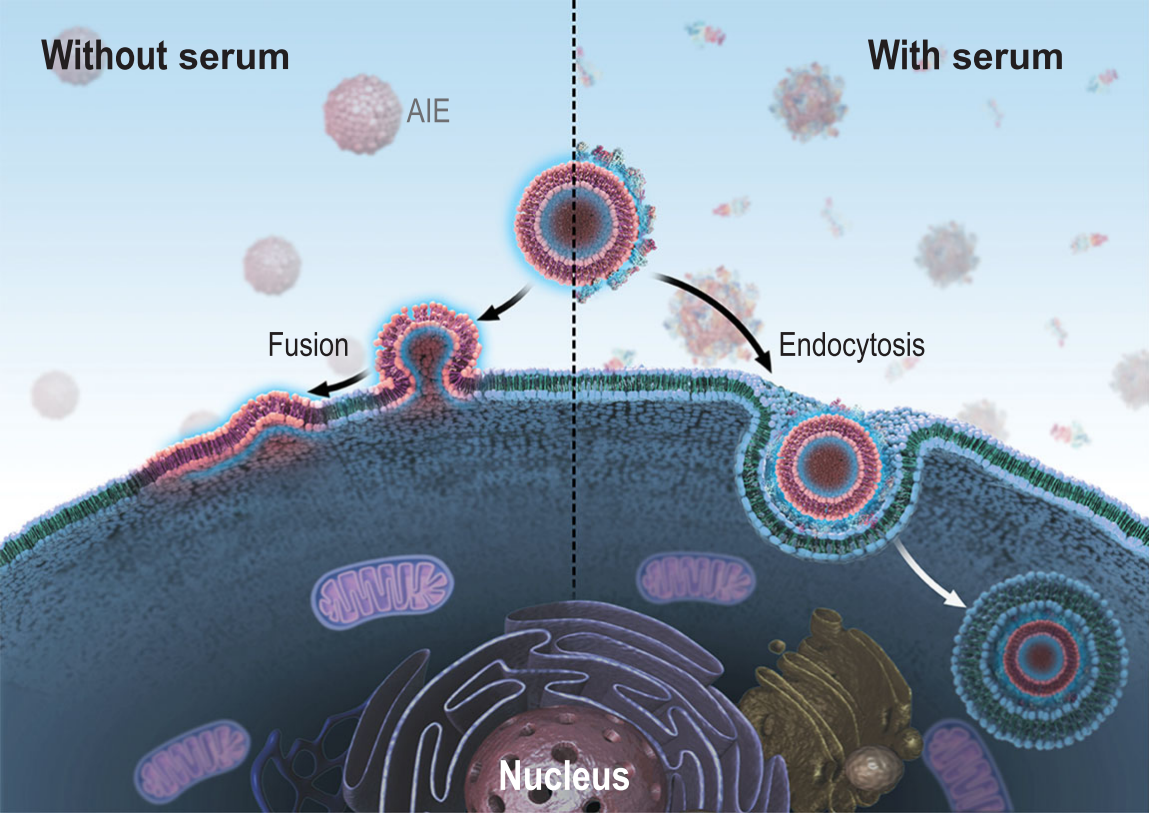
Transportation mechanism of cationic nanoliposomes, visualized by AIE, is dominated by the protein corona. In the absence of serum, the AIE-visualized liposomes interact with the cell membrane through fusion (left side). In the presence of serum, the AIE-visualized liposomes can be taken up by endocytosis (right side).

This is the first study to report that the protein corona can switch the interaction of cationic liposomes with cells from energy-independent membrane fusion to energy-dependent endocytosis. Once the nanostructures are incubated with biological fluids, assembly of biological components on their surface leads to a biological layer, which is known as the protein corona [16,19]. We employed a home-made AIE-visualized nanoliposome TR4@Lipo as a model to investigate the transportation mechanism with or without the protein corona. The results demonstrated that in the absence of serum, TR4@Lipo fuse with the cell membrane, but it is internalized through endocytosis when the protein corona forms on the surface (Scheme 1). Importantly, the intracellular distribution of loaded cargoes is also modulated accordingly by the formation of the protein corona. This knowledge furthers our understanding of bio-nano interaction, and is of particular practical significance for the efficient use of cationic liposomes for three reasons.

Firstly, our results clearly highlight the importance of selecting the proper conditions for an *in vitro* delivery system. As the final intracellular fate of small molecule drugs loaded in the nanocarriers determines the eventual therapeutic efficiency, the treatment effect of loaded cargoes will be potentially affected by the protein corona as we reported here. Thus, the delivery conditions should be carefully selected based on the loaded drug inside the cationic liposomes. For example, if the drug exerts its effect in lysosomes, it would be better to treat the cells in the presence of serum. However, if the loaded cargo exerts its effect in the nucleus, such as for gene delivery, serum-free conditions are the best choice. This is a good explanation of why the transfection efficiency of liposome-related reagents is higher in serum-free conditions than in the presence of FBS [47,48]. Secondly, similar considerations should be applied to cationic liposomes administered *in vivo*. Even though our *in vitro* evaluation was carried out using the standard serum concentration for cell culture (10% FBS), the critical serum concentration for switching from membrane fusion to endocytosis for some cationic liposomes could be higher *in vivo* than the standard serum concentration for cell culture as the protein concentration in serum is higher [49]. Thus, the transportation mechanism would be significantly different between the *in vivo* and *in vitro* situations even if the concentrations of liposomes are the same in the targeted organ and *in vitro*. Finally, our study shows the important influence of experimental conditions on conclusions, and suggests that the similarity of materials and protocols should be kept in mind when the conclusions from different labs are compared [50]. Overall, we believe that this insight into the effect of protein corona on the transportation mechanism of nanoliposomes will promote a better understanding of bio-nano interaction at the interface level and will guide the utilization of nanoliposomes in the future.

## METHODS AND MATERIALS

The experimental details are given in the online supplementary data.

## Supplementary Material

nwab068_Supplemental_File
