# Supplementary material for: Transportation of AIE-visualized nanoliposomes is dominated by the protein corona
Source: Natl Sci Rev. 2021 Apr 24;8(6):nwab068. doi: 10.1093/nsr/nwab068 (PMC8288177; doi:10.1093/nsr/nwab068)
Supplement: nwab068_Supplemental_File [file nwab068_supplemental_file.docx]

**Transportation of AIE-Visualized Nanoliposomes is Dominated by the Protein Corona**

Yi-Feng Wang^1,3,#^, Chunqiu Zhang^1,2,#^, Keni Yang^1,#^, Yufei Wang^1,3^, Shaobo Shan,^1^ Yan Yan^5,6^, Kenneth A. Dawson^4,5^, Chen Wang^1,3^, Xing-Jie Liang^1,3,^*

1. CAS Key Laboratory for Biomedical Effects of Nanomaterials and Nanosafety, CAS Center for Excellence in Nanoscience, National Center for Nanoscience and Technology of China, No. 11, First North Road, Zhongguancun, Beijing, 100190, P.R. China. Tel: +86-010-82545569; E-mail: [liangxj@nanoctr.cn](mailto:liangxj@nanoctr.cn).
2. State Key Laboratory of Medicinal Chemical Biology, Key Laboratory of Bioactive Materials, Ministry of Education, and College of Life Sciences, Nankai University, Tianjin 300071, China.
3. University of Chinese Academy of Sciences. Beijing 100049, P. R. China.
4. Guangdong Provincial Education Department Key Laboratory of Nano-Immunoregulation Tumor Microenvironment, The Second Affiliated Hospital, Guangzhou Medical University, Guangzhou, 510260 Guangdong, P.R. China.
5. Centre for BioNano Interactions, School of Chemistry, University College Dublin, Belfield, Dublin 4, Ireland.
6. School of Biomolecular and Biomedical Science, University College Dublin, Belfield, Dublin 4, Ireland.

#. These authors contributed equally.

KEYWORDS: aggregation-induced emission (AIE), cationic liposomes, protein corona, endocytosis, membrane fusion,

**Methods**

**Preparation of TR4@Lipo and normal Lipo, and DOX-loaded liposomes**

The TR4@Lipo was made by mixing HSPC, cholesterol, DSPE-PEG2000 and TR4 in a solvent mixture of chloroform/methanol (3/1). The ratio of TR4 to HSPC was varied from 1/100 to 1/25. The solvent was then evaporated to form a dry thin film on the flask. To induce liposome formation, water or PBS buffer was added into the flask and the flask was vibrated for 1 h at 37 ℃. The liposome solution was filtered through a 0.45 and 0.22 µm filter and then collected for characterization. The normal liposomes were made as above, but without adding TR4 at the beginning. To load a drug into liposomes, the dried lipid film was rehydrated by hydrophilic doxorubicin in PBS, followed by 1 h vibration at 37 ℃. The liposome solution was filtered through a 0.45 and 0.22 µm filter and the free doxorubicin was then removed by ultraspin filter.

**Cell culture**

The human breast cancer cell line MCF-7, the human adenocarcinoma cell line A549, the normal rat kidney cell line NRK, the human triple-negative breast cancer cell line MDA-MB-231 and the normal mouse muscle cell line C2C12 were purchased from ATCC. MCF-7, A549, NRK, MDA-MB-231 and C2C12 cells were maintained in DMEM medium with 10% FBS at 37 ℃.

**Uptake and intracellular distribution analysis**

Before the experiments, MCF-7, A549, NRK, MDA-MB-231 and C2C12 cells were seeded at a density of 3*10^4^ cells per well in 8-well chamber coverslips and incubated at 37 °C for 24 h. The cells were washed with PBS and then incubated with TR4@Lipo in different concentrations of FBS for 4 h at 37 °C. Then, the liposomes were removed and the cells were stained with LysoTracker Deep Red for 30 min at 37 °C. After that, the cells were washed again three times with PBS and imaged using a confocal microscope (LSM710, Carl Zeiss). The excitation was at 405 nm for TPE and at 630 nm for LysoTracker Deep Red. To test the effect of different serum proteins on the transportation mechanism of TR4@Lipo, human serum albumin and human serum were used to form a protein corona on the TR4@Lipo surface and then the uptake by MCF-7 cells was analyzed as described above.

To analyze the intracellular distribution of DOX in TR4@Lipo and normal Lipo, MCF-7 cells were incubated with free DOX, DOX@Lipo, or TRD@Lipo for 4 h at 37 °C. Then, cells were rinsed three times with PBS and incubated with LysoTracker Deep Red. After 30 min incubation, cells were washed three times with PBS and imaged using a confocal microscope (LSM710, Carl Zeiss). The excitation was at 405 nm for TPE, at 488 nm for DOX and at 630 nm for LysoTracker Deep Red. ImageJ software was used for co-localization analysis.

**Inhibition studies**

MCF-7 cells were seeded at a density of 1*10^5^ cells per well in a 12-well plate. After 24 h, the cells were washed three times with PBS and treated with the following inhibitors in serum-free media for 1 h at 37 °C: sodium azide (NaN_3_, 50 mM); the membrane fusion inhibitor analog Z-Phe-Phe-Phe-OH (50 μg/mL); and the endocytosis inhibitors chlorpromazine (Chl, 12.5 μg/mL), cytochalasin D (CytD, 5 μg/mL), and methyl-β-cyclodextrin (M-β-CD, 5 μg/mL). Afterwards, TR4@Lipo in the presence or absence of serum was added to the cells with or without the inhibitors for another 4 h. For the temperature-dependent pathway study, cells were washed three times with PBS and incubated for 1 h at 4 °C and then with TR4@Lipo in the presence or absence of serum for another 4 h. After that, cells were washed once with PBS, harvested using trypsin, washed again three times with PBS, and resuspended in PBS. Then, the median fluorescence intensity of the cells was determined with a flow cytometer (Attune NxT) at 405 nm. At least 10000 cells were recorded for each sample.

For inhibition studies followed by fluorescence microscopy, MCF-7 cells were seeded at a density of 3*10^4^ cells per well in 8-well chamber coverslips 24 h before the experiment. On the day of inhibition, cells were treated with inhibitors and TR4@Lipo in the absence or presence of FBS as described above, and then cells were washed three times with PBS and treated with LysoTracker Deep Red to stain lysosomes. After 30 min, cells were washed three times again with PBS and imaged using a confocal microscope (LSM710, Carl Zeiss). The excitation was at 405 nm for TPE and at 630 nm for LysoTracker Deep Red.

**Analysis of the protein corona**

100 µl of 20% (v/v) serum was added into 100 µl of liposomes. Then, the mixture was incubated with shaking at 200 rpm for 1 hour at 37 °C. To remove unbound serum proteins, the liposomes were pelleted by centrifugation at 22000 g for 40 mins at 4 °C and then the corona was washed twice with PBS. For the electrophoretic analysis, the samples were resuspended in SDS-PAGE loading buffer and sonicated for 5 mins. Then, the solutions were loaded into the gel after boiling for 10 mins at 100 °C. The proteins were separated at 80 mV for 5 mins and 120 mV until the loading buffer band reached the bottom of the gel. The gel was stained with Coomassie Brilliant Blue R250 for 1 h and then washed three times with a solution containing 20% ethanol and 10% acetic acid for 1 h, and pictures were taken using a ChemiDoc Touch Imaging System (BIO-RAD). For the LC-MS/MS analysis, the proteins were separated with 12% SDS-PAGE and then digested in-gel with trypsin. The peptides were analyzed by a Q Exactive mass spectrometer that was coupled to an EASY-nano LC system. The spectra were searched using the MASCOT engine. The mass tolerance was 20 ppm and MS/MS tolerance=0.1 Da. The UniProt database was searched for protein identification.

**Supporting Figures**


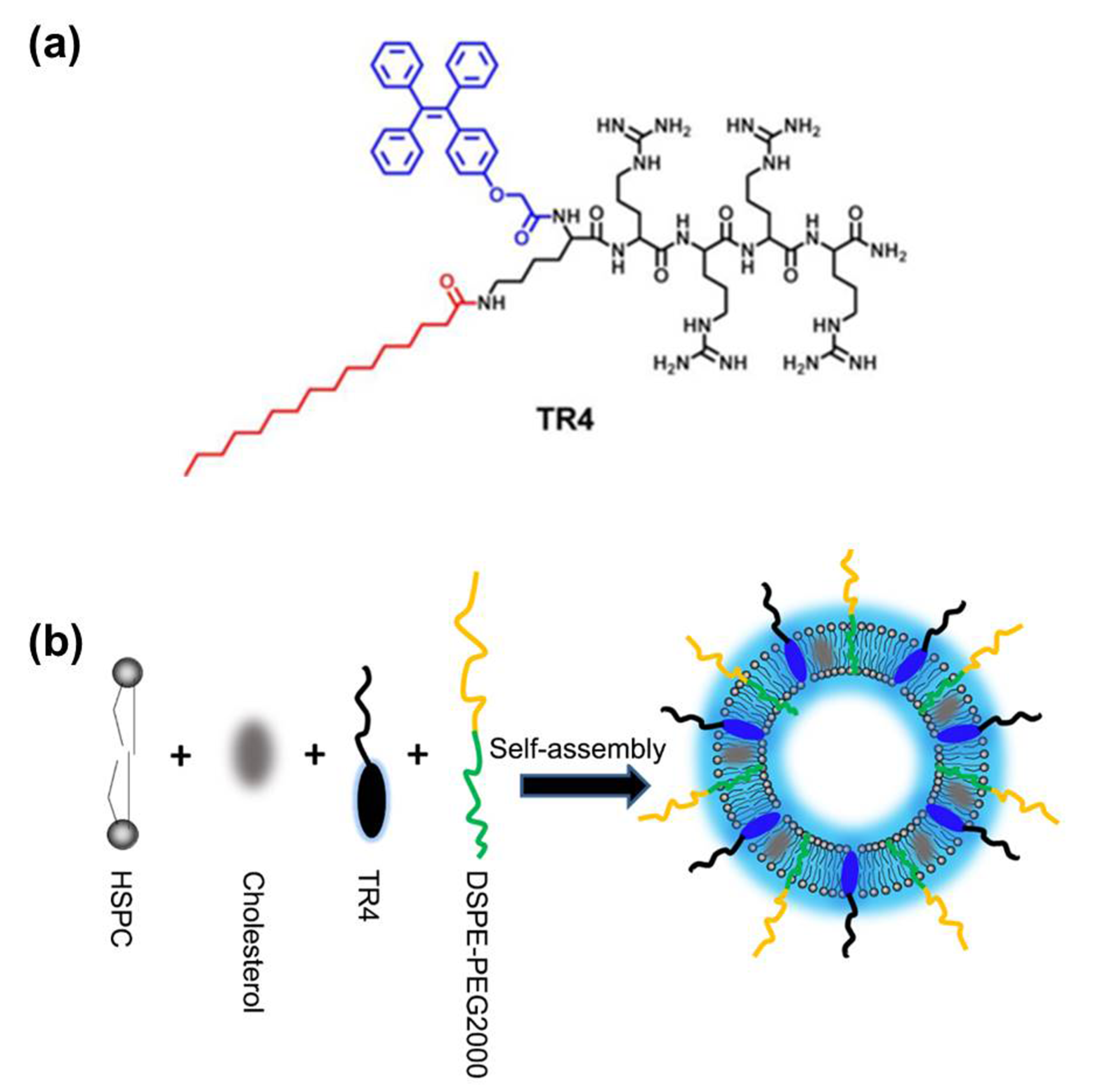


**Supporting Figure 1**. (a) Molecular structure of TR4. (b) Schematic illustration of the self-assembled structure of TR4@Lipo. TR4 generates blue fluorescence when incorporated into the liposomes.


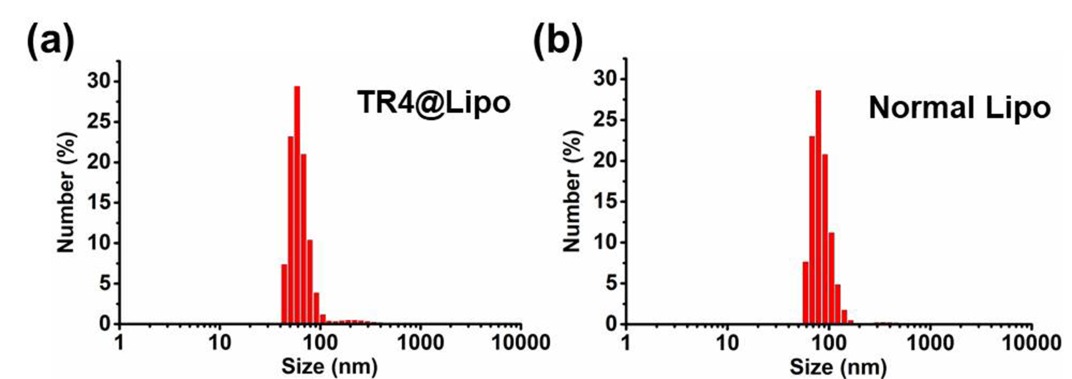


**Supporting Figure 2**. Size distribution by number of (a) TR4@Lipo and (b) normal Lipo.


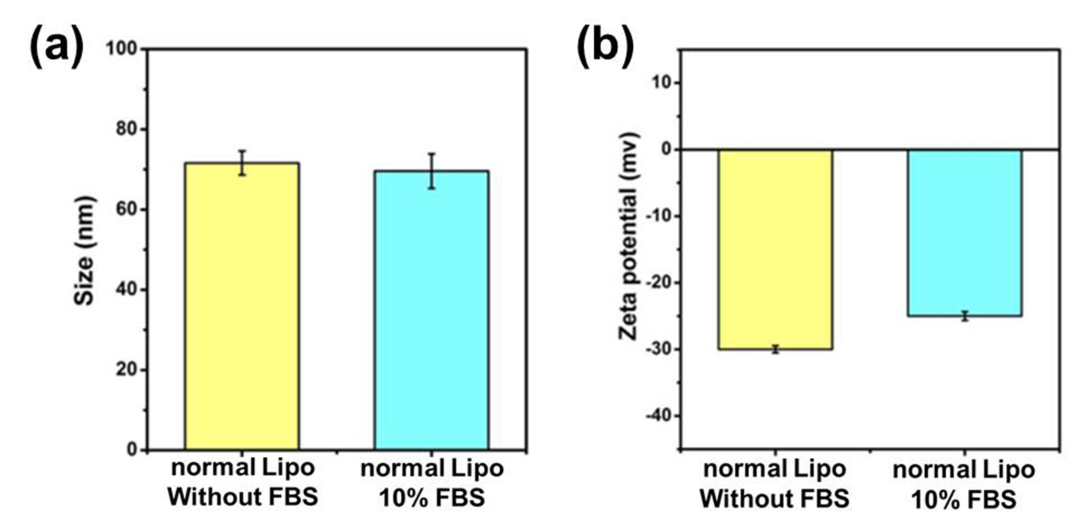


**Supporting Figure 3**. (a) Size and (b) zeta potential of normal Lipo in the presence or absence of 10% FBS.


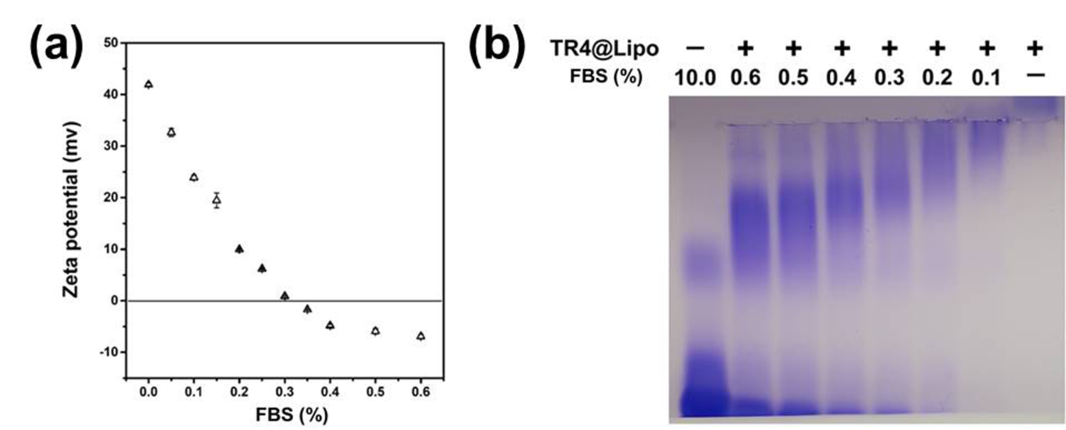


**Supporting Figure 4**. (a) Zeta potential of TR4@Lipo after incubation with different concentrations of FBS from 0.0 to 0.6%. (b) Non-denaturing gel electrophoresis analysis of liposome-bound serum proteins at the different FBS concentrations.


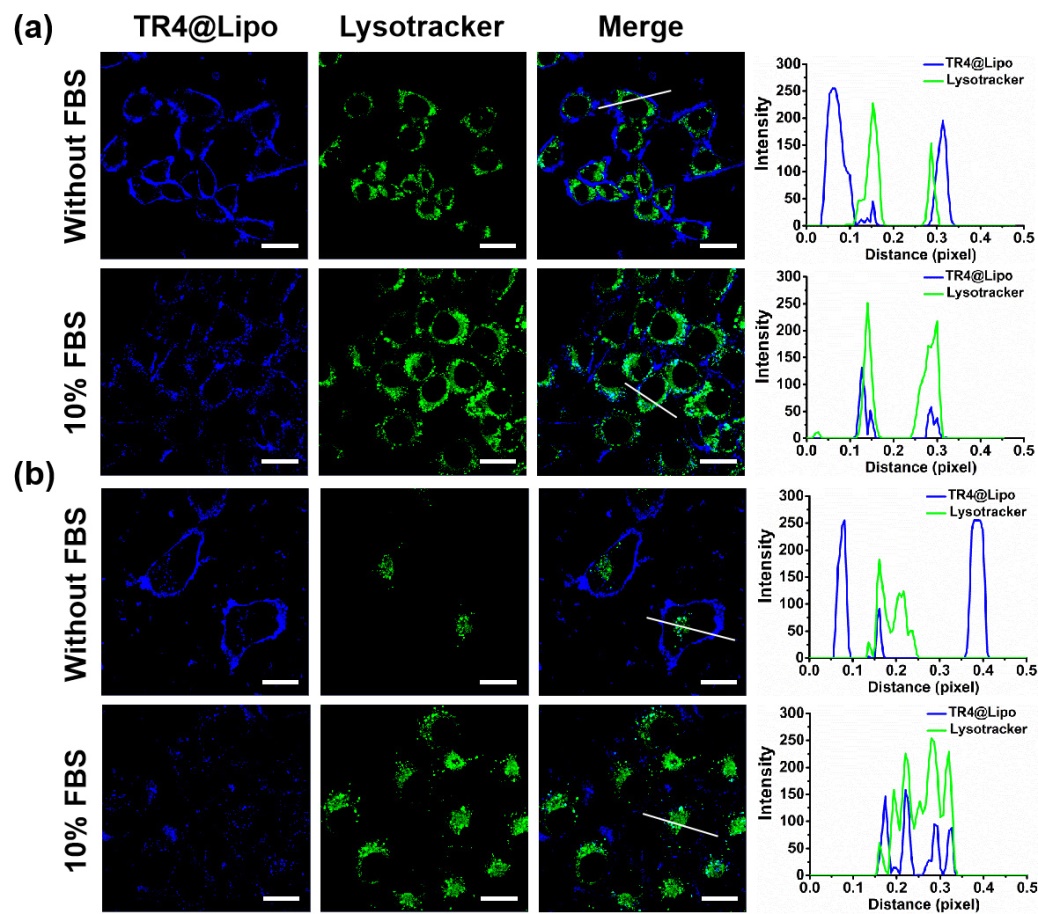


**Supporting Figure 5**. Confocal images of (a) the human triple-negative breast cancer cell line MDA-MB-231 and (b) the normal mouse muscle cell line C2C12 after treatment with TR4@Lipo in 10% FBS. Scale bar is 20 μm. Co-localization profiles, analyzed along the white lines in the “Merge” images, are shown at the right. The blue color is from TR4 (λex = 405 nm). The green color is from LysoTracker Deep Red (λex = 630 nm). ImageJ software was used for co-localization analysis.


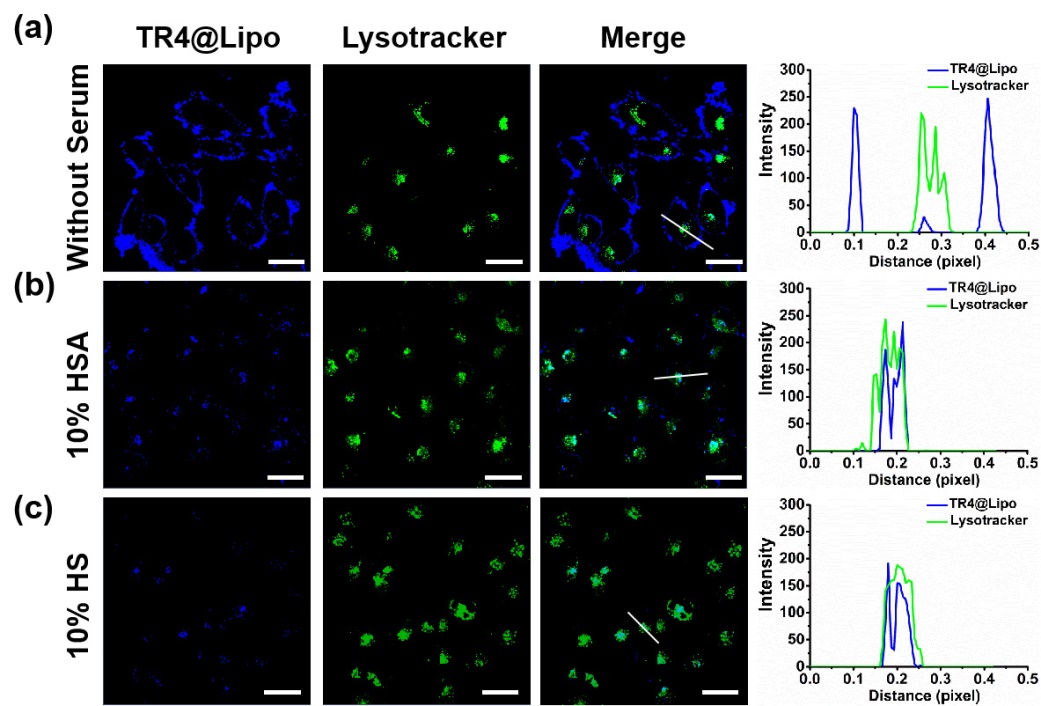


**Supporting Figure 6**. Confocal images of MCF-7 cells after treatment with TR4@Lipo (a) in the absence of serum, (b) in the presence of human serum albumin (HSA) and (c) in the presence of human serum (HS). Scale bar is 20 μm. Co-localization profiles are shown at the right and were analyzed along the white lines in the “Merge” images. The blue color is from TR4 (λex = 405 nm). The green color is from LysoTracker Deep Red (λex = 630 nm). ImageJ software was used for co-localization analysis.


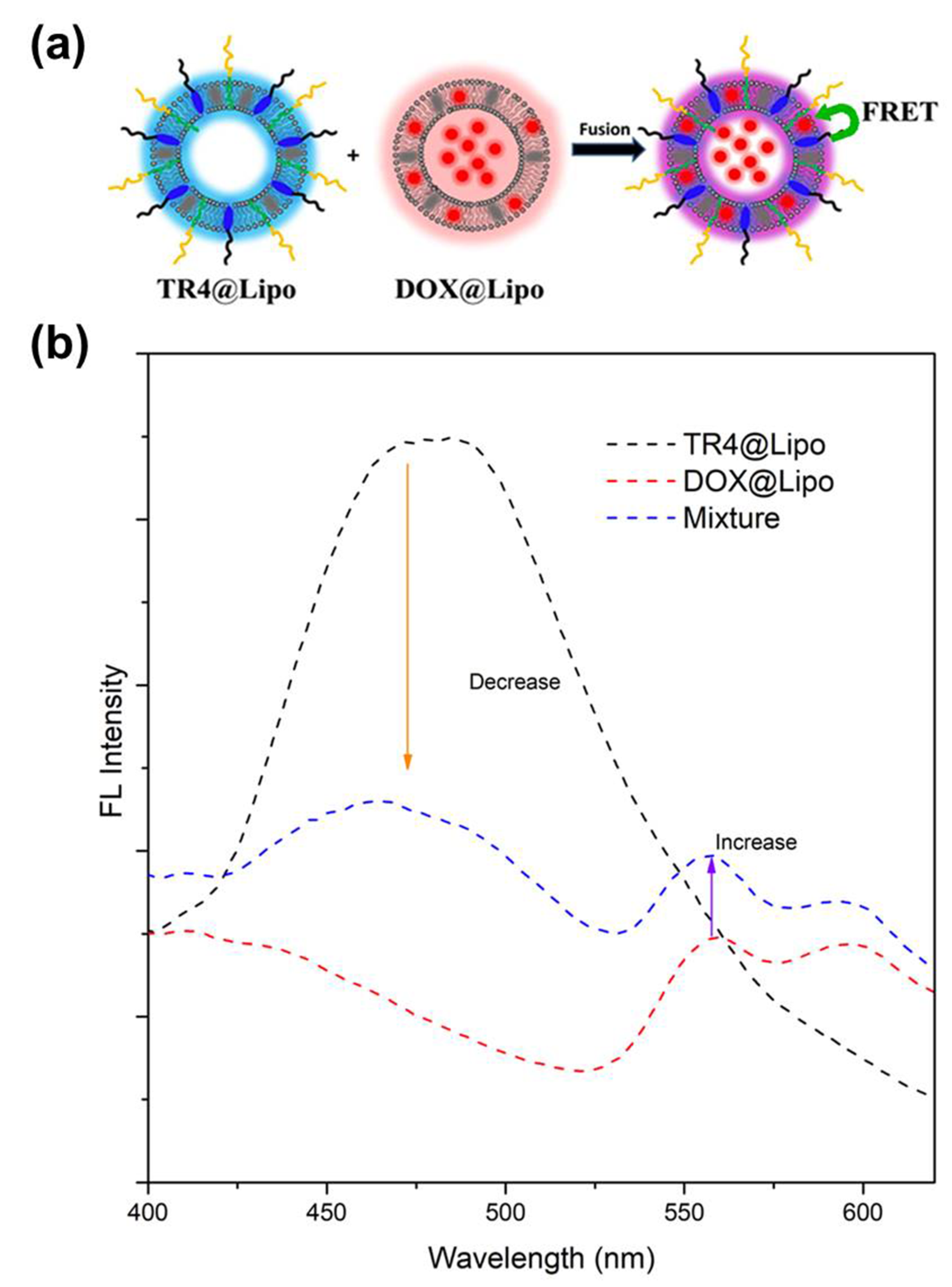


**Supporting Figure 7**. (a) Schematic illustration of the FRET model for TR4@Lipo and DOX@Lipo. (b) The fluorescence spectrum of TR4@Lipo, DOX@Lipo and a mixture of both. When the TR4@Lipo and DOX@Lipo were mixed together, the fluorescence intensity at 466 nm of TR4 was decreased and the intensity at 590 nm of DOX was simultaneously increased. This indicates that the energy of TR4 was transferred to DOX, as depicted in the FRET model in (a).





**Supporting Figure 8**. Uptake of TR4@Lipo by MCF-7 cells in the absence or presence of serum for 4 h under the following conditions: 12.5 μg/mL chlorpromazine (Chl); 5 μg/mL cytochalasin D (CytD); 5 μg/mL methyl-β-cyclodextrin (M-β-CD). Datasets were compared by unpaired t-test to identify significant differences. *, p < 0.05; ns, no significant inhibition.


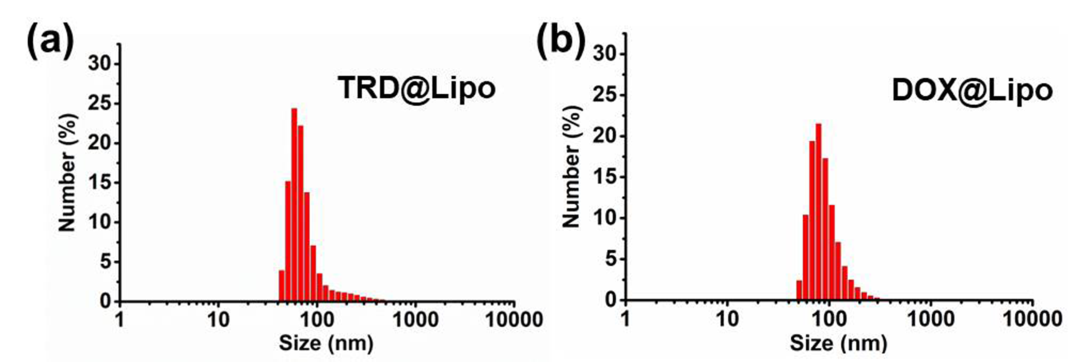


**Supporting Figure 9**. Size distribution by number of (a) TRD@Lipo and DOX@Lipo.


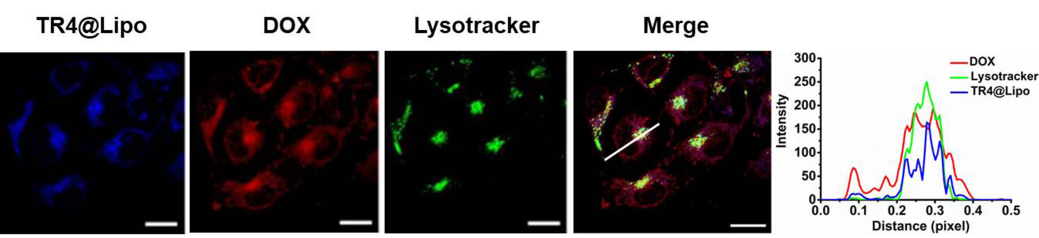


**Supporting Figure 10**. Confocal images depicting the subcellular distributions of TRD@Lipo which were coated with the protein corona and then incubated with MCF-7 cells in PBS buffer. The right panel shows co-localization analysis. Fluorescence intensities were measured along the white line in the “Merge” image. The blue color is from TPE (λex = 405 nm), the red color is from DOX (λex = 488 nm), and the green color is from LysoTracker Deep Red (λex = 630 nm). Scale bar is 20 μm. Co-localization analysis was performed with ImageJ.

**Supporting Table 1.** Dynamic light scattering analysis and zeta potential measurements of different liposomes.

**
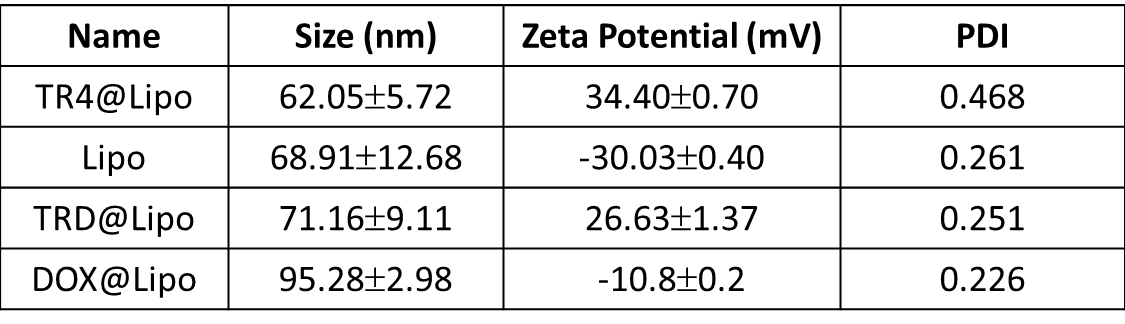
**

**Supporting Table 2.** Top 20 most-abundant proteins identified in the corona of TR4@Lipo and normal liposomes after incubation with mouse serum or FBS. TM, TR4@Lipo incubated with 10% mouse serum; LM, normal Lipo incubated with 10% mouse serum; TF, TR4@Lipo incubated with 10% FBS; LF, normal Lipo incubated with 10% FBS. The darker blue shading highlights 11 proteins that are among the top 20 for both TM and LM (10% mouse serum groups) and 7 proteins that are among the top 20 in both TF and LF (10% FBS groups).

**
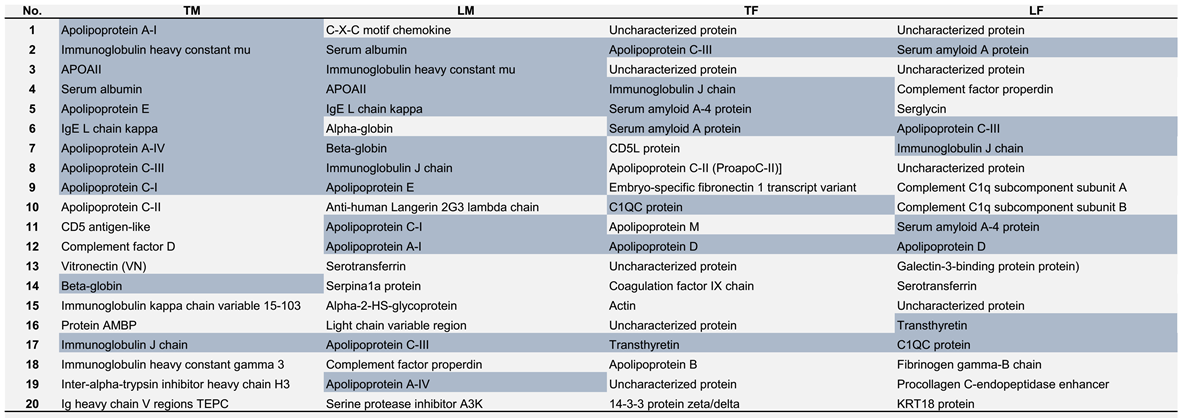
**
